# Analysis of morphological attributes as a driver of trade in poison dart frogs

**DOI:** 10.1111/cobi.70061

**Published:** 2025-05-31

**Authors:** Puja Jaichand, David L. Roberts, Iain M. Fraser

**Affiliations:** ^1^ Durrell Institute of Conservation and Ecology University of Kent Canterbury UK; ^2^ School of Economics, Politics, and International Relations University of Kent Canterbury UK

**Keywords:** exotic pets, hedonic analysis, online trade, preference, wildlife trade, análisis hedónico, mascotas exóticas, mercado de fauna, mercado en línea, preferencia

## Abstract

The unsustainable use of wildlife is a threat to biodiversity on a global scale, and the insatiable demand is driven by the attributes of the species, their parts, and derivatives. However, not all species are equally valued; certain attributes command a higher price. One example is the exotic pet trade in amphibians and reptiles. Poison dart frogs (Dendrobatidae) are particularly in demand owing to their vibrant colors and diurnal behavior. Focusing on the dart frog genus *Dendrobates*, we examined buyer preference for specific attributes. For this, we collected market data from the online trade of *Dendrobates* and combined these with morphological data. The attribute data collected from online platforms included species, locale, country sold from, company or platform, origin, sex, size, and age. These data were combined with morphological attribute data for each species and locale. We used hedonic price regression analysis to ascertain whether the selected attributes influenced the price of dart frogs. Species, age, region sold from, market rarity, head color, and trunk colors were all predictors of price. With such knowledge, it may be possible to prioritize those species in particular demand to increase the economic return to range states and local communities through livelihood initiatives. Further, it may be possible to anticipate the value of newly described species and identify those that may become threatened through unsustainable trade.

## Abstract (es)

Análisis de los rasgos morfológicos como factores en el mercado de ranas venenosas

**Resumen:**

El uso insostenible de fauna es una amenaza para la biodiversidad a escala mundial, y la insaciable demanda está impulsada por los rasgos de las especies, sus partes y derivados. Sin embargo, no todas las especies tienen el mismo valor; algunos atributos tienen un precio más alto. Un ejemplo es el comercio de anfibios y reptiles exóticos como mascotas. Las ranas venenosas (Dendrobatidae) están especialmente solicitadas por sus vivos colores y su comportamiento diurno. Nos enfocamos en el género de ranas dardo *Dendrobates* y analizamos las preferencias de los compradores por atributos específicos. Para ello, recopilamos datos de mercado del comercio en línea de *Dendrobates* y los combinamos con datos morfológicos. Los datos de rasgos recogidos de las plataformas en línea incluían la especie, la localidad, el país desde el que se vende, la empresa o plataforma, el origen, el sexo, el tamaño y la edad. Estos datos se combinaron con datos de rasgos morfológicos para cada especie y localidad. Usamos un análisis de regresión hedónica de precios para determinar si los atributos seleccionados influían en el precio de las ranas dardo. La especie, la edad, la región de venta, la rareza del mercado, el color de la cabeza y del tronco fueron factores predictivos del precio. Con estos conocimientos, puede ser posible dar prioridad a las especies con mayor demanda para aumentar el rendimiento económico de los países del área de distribución y las comunidades locales a través de iniciativas de subsistencia. Además, quizá sea posible anticipar el valor de las especies recién descritas e identificar las que pueden verse amenazadas por un comercio insostenible.

## INTRODUCTION

The wildlife trade is a lucrative industry. From 1997 to 2016, its value was thought to be US$2.9–4.4 trillion (Andersson et al., 2021). This trade in animals, plants, their parts, and derivatives has been steadily increasing, especially with the shift to online trade, and can be highly unsustainable when left unregulated (Harrison et al., 2016). Wildlife trade is driven by the demand for food, medicine, clothing, and exotic pets, among other factors (Hughes, 2021). The exotic pet trade, defined as the trade in those species that are not typically domesticated (Price, 1984), is now more prevalent than ever, with the growing popularity of online trade and an increase in consumer numbers (Bush et al., 2014; Harrison et al., 2016). Amphibians are one of the most affected vertebrate groups, both directly and indirectly, by the exotic pet trade; nearly 20% of recorded species are threatened with extinction (Ceballos et al., 2020). Overharvesting has been linked to a decline in wild populations, yet trade is largely unmonitored and unregulated for approximately 98% of amphibian species (Auliya et al., 2016; Kaczmarski & Kolenda, 2018).

Poison dart frogs, also referred to as poison arrow frogs (family Dendrobatidae), from Central and South America constitute a significant proportion of the amphibians in the exotic pet trade. Over 180,000 Dendrobatidae spp. were recorded as being traded from 2000 to 2014 (Auliya et al., 2016; Edmonds, 2021b; Kaczmarski & Kolenda, 2018; Mohanty & Measey, 2019; Nijman & Shepherd, 2010; Yeager & Zarling, 2020). Their popularity is due to their attributes, such as bright coloration, size, and diurnal behavior (Mohanty & Measey, 2019). Although all species in the genus *Dendrobates* are listed as least concern according to the IUCN Red List, they would benefit from monitoring due to the sheer amount of trade (Edmonds, 2021a; IUCN, 2022). Some species, such as the green and black dart frog (*Dendrobates auratus*), have already shown global population declines (IUCN, 2022). A study undertaken by Kaczmarski and Kolenda (2018) in Poland showed that dart frogs are often the most prevalent in online shops, comprising 46% of the amphibians in the sample, and a global study by Mohanty and Measey (2019) found a strong bias toward the Dendrobatidae.

Due to concerns of unregulated trade threatening their populations, the Dendrobatidae family was added to Appendix II of the Convention on the International Trade in Endangered Species of Fauna and Flora (CITES) in an effort to regulate trade in 1987 (Gorzula, 1996). Appendix II pertains to species that are not immediately at risk of extinction but may become so if trade is not monitored or regulated. As a result, export permits are required for any international trade, although some states have stricter measures requiring an additional import permit (CITES, 2022). Despite this, illegal trade persists with notable discrepancies between export and import numbers in certain trade routes (Nijman & Shephard, 2010). To curb illegal trade, organizations (e.g., Tesoros de Colombia) have been established in range countries, such as Peru, Colombia, and Ecuador, with the purpose of establishing ex situ breeding facilities to supply this market demand (Edmonds, 2021a, 2021b; Yeung, 2020). This method meets consumer demand with legally bred frogs while simultaneously reducing the demand for wild caught frogs. However, due to the level of consumer demand, others have established breeding facilities outside of range states, with an estimated 100,000 breeders in the United States alone (Edmonds, 2021a, 2021b).

Market analyses of consumer choice with both stated and revealed methodologies show that variation in individual preferences is a major driver in all aspects of wildlife trade (Sung & Fong, 2018). Consumer preferences tend toward rarer species, whether due to their unusual morphological attributes, their frequency on the market, or rarity in the wild (Hinsley et al., 2015; Krishna et al., 2019; Lyons & Natusch, 2013; Sung & Fong, 2018). Krishna et al. (2019) found a positive correlation between rarity and market price for caged birds in Sumatra, where rarity on the market was highly valued by consumers, whereas rarity in the wild was irrelevant if the species was frequently encountered in markets. Lyons and Natusch (2013) likewise showed a positive correlation between price and preference for green pythons (*Morelia viridis*) from populations considered rare due to coloration. Sung and Fong (2018) also found that price of turtles increases based on the rarity of species; rarity was based on their IUCN status or any special morphological attributes like albinism. When a link between rarity and price exists, it can result in a threat to the species and thus the anthropogenic Allee effect (AAE). The AAE predicts that species rarity and its value are positively correlated, which leads to an increase in demand and thus overexploitation of a species (Courchamp et al., 2006).

The online global trade of wildlife has become one of the biggest threats to biodiversity, alongside other factors, such as habitat destruction and the spread of diseases, due to its sheer scope and scale (Di Minin et al., 2019). The ever‐increasing ease of accessing the internet has provided countless opportunities for both legal and illegal wildlife trade through social media, online forums, and marketplaces. At the same time, it provides an opportunity to collect and use online data to assess trends in the wildlife trade. Using price data from online platforms, we aimed to determine drivers of demand in the wildlife trade in a case study of dart frogs in the genus *Dendrobates*. Using hedonic price models, which are frequently employed in economics (Gibbons et al., 2014; Núñez et al., 2024), we focused on the demand for morphological attributes, such as color and patterning. Understanding the drivers of demand will not only allow conservationists to better prioritize interventions, such as wildlife use through sustainable livelihood models, but also identify the value of new species and the extent to which they are likely to be threatened by trade.

## METHODS

### Ethics

Ethical approval was received from the Research and Ethics Committee of the School of Anthropology and Conservation, University of Kent, prior to the start of data collection. For ethical reasons, we did not believe it was justifiable to access closed groups given the nature of the study and the potential need to use deception (Thompson et al., 2021) because we did not seek to examine illegal trade or to conduct a global analysis.

### Data collection

We collected data on *Dendrobates* available for sale from 18 June 2022 to 16 July 2022. A preliminary Google search was conducted on 17 June 2022 to identify any potential attributes that could affect the price. The attributes identified were species, locale, the country sold, company or platform, origin (captive bred or wild caught), sex (male or female), size (millimeters), and age (juvenile or adult). These were further corroborated through informal discussions with 2 keepers and a trader.

The Google search function was used to conduct a systematic search for 2 key terms: “*Dendrobates sale*” and “*Dendrobates price*” (Roberts et al., 2022). The uniform resource locators (URLs) regarding *Dendrobates* trade in the first 100 search results were recorded. This was done for both search terms, and duplicates were removed. Information was collected from each URL as well as the website it was linked to by searching *Dendrobates* with the search option or by otherwise exploring the website. Data were collected from English‐language websites only, listing frogs that were in stock, as well as out of stock (tadpoles were excluded), resulting in an initial total of 420 data entries (Appendix S1). Because only open‐access platforms were searched, closed groups were not included.

Once data collection was completed, we converted all price data to United States dollars (US$). This conversion was undertaken using www.xe.com on 16 July 2022. The initial data set was cleaned by removing entries that lacked sufficient information, excluding entries that were incorrectly labeled, and calculating the price per individual frog for entries with multiple frogs. Once the data were cleaned, a separate Excel document was used to create a morphological data set for all species and locales in the collected sample with a search of Google Images. The morphological attributes were deduced by searching the complete species name (and locale if available) for each unique taxon. The morphological attributes that were recorded were head color, trunk or abdomen color, limb color, and pattern. Trunk color was separated into primary and secondary color, which we defined as the most prominent and least prominent colors present on the abdominal section of the frog. Pattern was also separated into 4 categories: rings, spots, stripes, and none.

### Data analyses

The number of attributes and their associated levels were grouped and aggregated where appropriate (Table 1). The price data and morphological data were then combined to produce the final set of variables (Table 2). Price as is common in cross‐sectional economic data exhibited a significant positive skew. In keeping with the hedonic price literature, we transformed the data by taking the natural log and employing this as our dependent variable. In terms of dependent variables, species was dummy coded as 1 for a specific species and 0 otherwise. Country was coded as 1 if being sold in Europe and 0 if in North America. Head color and limb color were coded as being the same as (0) or different from (1) trunk color. The individual colors under primary trunk color were grouped as black and not black, with the latter comprising blue, brown, cream, green, white, and yellow. Secondary trunk color was grouped as common (colors representing >18% of the sample) and rare (colors representing <18% of the sample). Common secondary trunk colors were black, blue, green, and yellow, and rare secondary trunk colors were brown, cream, white, and none. In addition, we constructed all the color combinations for the trunk (primary and secondary) for use as potential explanatory variables (Appendix S2). The use of this variable in model estimation did not improve model performance. Age was coded as 1 if an adult and 0 if a juvenile. Pattern was dummy coded as 1 for specific type of marking and 0 otherwise. Rarity was defined as the rarity on the market, that is, the frequency of the species in the collected data set (the lower the number of recorded species for sale, the rarer the species), rather than rarity in the wild. In the analyses, we employed the natural log of rarity to enable the derivation of the elasticity of rarity and to account for the negative skew in the data. Finally, due to data limitations, both sex and size were excluded from our analyses. We used NLOGIT 6 (https://www.limdep.com/products/nlogit/) to estimate all hedonic model specifications, including ordinary least squares (OLS) and random effects (RE) on the final data set.

**TABLE 1:** Grouping of specific attributes for data analyses in a study of dart frogs.

Variable	Attribute before grouping	Attribute after grouping
Age	Froglet	Juvenile
	Juvenile	Juvenile
	Subadult	Adult
	Adult	Adult
Country sold	Belgium	Europe
	Canada	North America
	United Kingdom	Europe
	United States	North America
Head color	Listed by individual colors	Same or different from trunk color
Trunk color (primary)	Listed by individual colors	Black or not black
Trunk color (secondary)	Listed by individual colors	Common or rare
Limb color	Listed by individual colors	Same or different from trunk color

**TABLE 2:** List of variables in the final data set in an examination of pricing in dart frogs.

Variable type	Variable name (Label)	Unit
Price (log)	Lnprice	U.S. dollars
Species	*Auratus* *Leucomelas* *Tinctorius* *Truncatus* ^a^	Indicator variable
Age	Adult ( = 1) Juvenile ( = 0)	Binary variable
Country sold	Europe ( = 1) North America ( = 0)	Binary variable
Rarity within sample (log)	Ln rarity	Number
Head color	Head color ( = 1) No head color ( = 0)	Binary variable
Abdomen or trunk color (primary)	Black ( = 1) Not black ( = 0)	Binary variable
Abdomen or trunk color (secondary)	Common ( = 1) Rare ( = 0)	Binary variable
Color of limbs	Limb color ( = 1) No limb color ( = 0)	Binary variable
Pattern	Rings Spots Stripes None^a^	Indicator variable

^a^ Dummy variable level excluded in regression analyses to avoid dummy variable trap.

## RESULTS

A total of 420 individual data points were initially collected. This number was reduced to 331 data points once entries that had age missing were removed (Table 3). These were collected from 21 different online platforms that ranged from websites specifically dedicated to exotic pet sales to individual retailers on varying sale sites. These websites were distributed among 4 separate countries: Belgium, Canada, the United Kingdom, and the United States. The latter had the most data points, accounting for 79%. A total of 83 distinct taxa (including locales and morphs) were recorded. Median rarity on the market was 8 (range 1–35). *Dendrobates leucomelas* and *Dendrobates tinctorius azureus* were the most common. Prices ranged from US$19.67 to US$399.50 per individual frogs; the median was US$65.18. With regard to the data set defined by age, adult frogs cost approximately 60% more than juvenile frogs.

**TABLE 3:** Statistical summary of attributes in the data set (*n* = 420) in an examination of pricing in dart frogs.

Attribute	Attribute categories	Statistical summary
Price	–	Range US$19.67–399.50 Median US$65.18
Species % (*n*)	*Auratus*	33.3 (140)
	*Leucomelas*	12.9 (54)
	*Tinctorius*	53.3 (224)
	*Truncatus*	0.5 (2)
Age % (*n*)	Adult	12.7 (42)
	Juvenile	87.3 (289)
Country sold % (*n*)	Belgium	3.3 (14)
	Canada	2.6 (11)
	United Kingdom	15.5 (65)
	United States	78.6 (330)
Rarity	–	Range 1–35 Median 8
Head color % (*n*)	Same as trunk color	85.7 (360)
	Different from trunk color	14.3 (60)
Trunk color (primary) % (*n*)	Black	62.4 (262)
	Not black	37.6 (158)
Trunk color (secondary) % (*n*)	Common	87.1 (366)
	Rare	12.9 (54)
Limb color %	Same as trunk color	63.6 (267)
	Different from trunk color	36.4 (153)
Pattern % (*n*)	Rings	17.6 (74)
	Spots	15.7 (66)
	Stripes	63.1 (265)
	None	3.6 (15)

Our preferred hedonic price model specification (model 3) was selected (Table 4). This model 3 yielded a log likelihood of −10.37 and an AIC of 56.744. This model had multiple explanatory variables, including age and sample of *n* = 331 (Table 5). The exclusion of age, although it significantly increased sample size, had a negative impact on model performance (Table 4) across all model specifications examined.

**TABLE 4:** Model diagnostics and model selection results based on the price of dart frogs.

Model	Model type	Sample size^a^	Number of explanatory variables	Log likelihood (LL)	AIC^b^	Adjusted *R* ^2^
Model 1	Ordinary least squares	331	13	−18.17	62.74	0.31
Model 2	Ordinary least squares (OLS)	420	12	−119.14	262.28	0.07
Model 3	Random effects without correlated effects	331	18	−10.37	56.74	N/A
Model 4	Random effects without correlated effects	420	17	−113.18	260.4	N/A
Model 5	Random effects with correlated effects	331	21	−9.56	61.12	N/A
Model 6	Random effects with correlated effects	420	20	−111.85	263.7	N/A

^a^ Full sample (*n* = 420) are models that exclude explanatory variable adult, and subsample (*n* = 331) are models that include adult.

^b^ Akaike information criterion = 2 × k − 2 × LL.

**TABLE 5:** Preferred model specification for relationship between log of price and explanatory variables based on the price of dart frogs (*n* = 331).

Variable	Coefficient	SE	*p*
Fixed coefficients			
Intercept	4.016^b^	0.183	<0.001
Age	0.448^b^	0.031	<0.001
Country	−0.165^b^	0.037	<0.001
Ln rarity	−0.065^b^	0.011	<0.001
Head color	0.174^b^	0.032	<0.001
Black	−0.045^c^	0.024	0.064
Common	−0.169^b^	0.029	<0.01
Limb color	0.029	0.030	0.320
Rings	0.013	0.046	0.773
Spots	−0.042	0.027	0.121
Stripes	−0.012	0.027	0.634
Random coefficients (means)^a^			
*Auratus*	0.422^d^	0.181	0.020
*Leucomelas*	0.549^b^	0.189	0.004
*Tinctorius*	0.451^d^	0.182	0.013
Random coefficients (SD)			
*Auratus*	0.243^b^	0.017	<0.001
*Leucomelas*	0.236^b^	0.023	<0.001
*Tinctorius*	0.126^b^	0.012	<0.001
Log likelihood	−10.375		
Akaike information criterion	56.744		

^a^ All random coefficients assumed normal. Number of regressors = 18.

^b^ *p *< 0.01.

^c^ *p* < 0.05.

^d^ *p* < 0.1.

The RE variables for species type were all significant (*p* < 0.001) for both mean and standard deviation relative to the excluded species *Truncatus*. Thus, species type did not have a statistically significant influence on the price level observed in the market. For our fixed model parameters, age was prominent in the model with a positive coefficient and a *p* < 0.001. This indicated that the sale of older (Adult) animals attracted a higher price than juveniles. Country had a significant effect (*p* < 0.001) and negative coefficient, indicating that sale prices in Europe were lower than in North America. The natural log of rarity was significant (*p* < 0.001) and had a negative coefficient. This coefficient is an elasticity and indicated that species rarity on the market was extremely inelastic, which implies that rarity would need to increase significantly for there to be a reduction in price. Head color was significant (*p* < 0.001) and had a positive coefficient, which means species with a specific head color attracted a higher price. In terms of truck colors, both attributes had negative coefficients, but black was only statistically significant at *p* < 0.1, whereas common was significant at *p* < 0.001. The remaining explanatory variables (limb color, rings, spots, and stripes) did not yield statistically significant parameter estimates.

## DISCUSSION

Our study is one of the first to look at revealed consumer preferences for morphological attributes with a specific focus on color. A hedonic price regression analysis resulted in several attributes being significant predictors of price. The inclusion of age as a variable significantly improved regression model performance. Being an adult was positively and statistically significant in terms of explaining price variation. This could be due to the proportional relationship between the size of these frogs and their age, whether adult or juvenile (Edmonds, 2021a, 2021b). In this genus, adults tend to be larger than juveniles and can potentially be used for breeding. This would be more likely to result in a positive financial return on an investment. Further, dart frogs are easier to sex when they are adults, which was evident from the fact that most of the frogs in the data set were unsexed because they were juveniles. Therefore, buyers rely on chance if they are purchasing a juvenile frog with the intention of obtaining a specific sex. Adult frogs are also sexually mature and can be used for breeding; these are all traits that could make them more attractive to a potential consumer and thus value them higher in terms of price (Edmonds, 2021a, 2021b). A number of websites sold them as breeding pairs without the option of purchasing singular frogs.

Country was also highly significant. Its negative coefficient for Europe meant that the price of frogs in Europe was lower compared with North America. This variable was composed of data from Belgium and the United Kingdom. The significance here can be attributed to the exchange rate as well as availability. Interestingly, although only 19% of the frogs in the data set were being marketed in Europe, prices remained on the lower end.

In terms of color, head color was positively, statistically significant and was coded as the same as or different from trunk color. Due to head color having a positive coefficient, it suggests that frogs that had a different head color were priced higher than those that did not. This could be attributed to these frogs being more noticeable due to this feature because head colors are often more vibrant than trunk colors. Most sale descriptions placed emphasis on frogs with limbs that differed in color from the trunk, this variable was significant in the model. In terms of trunk color, although both primary (positive) and secondary (negative) trunk colors were statistically significant, primary was relatively weak. The negative coefficient on secondary trunk colors supports the perception that dart frogs are being collected for their colors (Mohanty & Measey, 2019).

Finally, rarity was negatively statistically significant, which is as we expected a priori. Hobbyists and collectors in this type of market seek the rarest species, and because rare species attract more buyers, they tend to be more expensive (Angulo & Courchamp, 2009; Bush et al., 2014; Courchamp et al., 2006; Slone et al., 1997).

Future studies regarding consumer preferences in the wildlife trade should consider the following. First, the lack of data pertaining to the genus being studied. Most notably, sellers did not provide information on attributes such as age, sex, and size of the frogs. There was a distinct lack of standardization in the information being offered to the consumer. A small portion of sellers listed all the information necessary, whereas others listed only some, and yet for some sale listings, only the species name was available. This greatly reduced the data set that a regression model could be based on. It is recommended that further research in this area should strive for a larger data set by extending the limit on search results and, if possible, by including more attributes. Second, some sellers still use outdated names and classifications for some frogs; some species also have multiple common names. This can cause confusion in the data set by duplicating species if not properly inspected and corrected. Because most of the frogs in the genus *Dendrobates* are commonly found in national and international trade, it would be prudent to repeat this study on a larger scale with the other related genera, such as *Adelphobates*, *Ameegra*, and *Oophaga*. This could lead to identifying which genus is more likely to be targeted for trade.

The results of our study go some way toward the identification of species that are more likely to be affected by trade, as well as species of high value that could result in increased economic return to range states and local communities. Informed legal biocommerce can be successful in supplanting illegal trade, as is evidenced by organizations such as Tesoros de Colombia. Further, it may also help predict the value and thus potential threat through trade should a new species be discovered and introduced to the market. Finally, it allows for monitoring and other regulations, such as harvest quotas for range states, to be put in place by the relevant organizations to ensure sustainable trade. Although *Dendrobates* populations are not currently at risk of extinction, according to the IUCN Red List, the frequency at which they are encountered in trade and their decreasing populations calls for monitoring and regulation as preemptive actions (IUCN, 2022; Nijman & Shepherd, 2010).

Market‐level data are critical for establishing effective conservation measures for traded species. As a case study of a wider issue of preference for certain morphological attributes (particularly color), our results suggest color is not a major driver of preference, rather it is the rarity of a given morphological attribute. However, because the wildlife trade is likely to be dynamic, with trends changing over time, it is important to identify those attributes that are significant in driving wildlife trade in order to ensure a sustainable and regulated trade.

## Supporting information



Appendix S1 – Summary of online data collected (n=420)

Appendix S2 – Primary and secondary trunk colour combinations (n=420)

Appendix S3 – Hedonic regression on the entire data set

## References

[cobi70061-bib-0001] Andersson, A. A. , Tilley, H. B. , Lau, W. , Dudgeon, D. , Bonebrake, T. C. , & Dingle, C. (2021). CITES and beyond: Illuminating 20 years of global, legal wildlife trade. Global Ecology and Conservation, 26, Article e01455.

[cobi70061-bib-0002] Angulo, E. , & Courchamp, F. (2009). Rare species are valued big time. PLoS ONE, 4(4), Article e5215. 19384416 10.1371/journal.pone.0005215 PMC2668182

[cobi70061-bib-0003] Auliya, M. , García‐Moreno, J. , Schmidt, B. R. , Schmeller, D. S. , Hoogmoed, M. S. , Fisher, M. C. , Pasmans, F. , Henle, K. , Bickford, D. , & Martel, A. (2016). The global amphibian trade flows through Europe: The need for enforcing and improving legislation. Biodiversity and Conservation, 25(13), 2581–2595.

[cobi70061-bib-0004] Bush, E. R. , Baker, S. E. , & MacDonald, D. W. (2014). Global trade in exotic pets 2006–2012: Exotic pet trade. Conservation Biology, 28(3), 663–676. 24661260 10.1111/cobi.12240

[cobi70061-bib-0005] Ceballos, G. , Ehrlich, P. R. , & Raven, P. H. (2020). Vertebrates on the brink as indicators of biological annihilation and the sixth mass extinction. Proceedings of the National Academy of Sciences of the United States of America, 117(24), 13596–13602. 32482862 10.1073/pnas.1922686117 PMC7306750

[cobi70061-bib-0006] Convention on International Trade in Endangered Species of Wild Fauna and Flora (CITES) . (2022). The CITES Appendices . https://cites.org/eng/app/index.php 10.1159/000459796 712806

[cobi70061-bib-0007] Courchamp, F. , Angulo, E. , Rivalan, P. , Hall, R. J. , Signoret, L. , Bull, L. , & Meinard, Y. (2006). Rarity value and species extinction: The Anthropogenic Allee effect. PLoS Biology, 4(12), Article e415. 17132047 10.1371/journal.pbio.0040415 PMC1661683

[cobi70061-bib-0008] Di Minin, E. , Fink, C. , Hiippala, T. , & Tenkanen, H. (2019). A framework for investigating illegal wildlife trade on social media with machine learning. Conservation Biology, 33(1), 210–213. 29528136 10.1111/cobi.13104 PMC7379580

[cobi70061-bib-0009] Edmonds, D. (2021a). Data for poison frogs in U.S. collections (Version 1) [Data set]. University of Illinois at Urbana‐Champaign. 10.13012/B2IDB-4717502_V1

[cobi70061-bib-0010] Edmonds, D. (2021b). Poison frogs traded and maintained by US private breeders. Herpetological Review, 52, 779–786.

[cobi70061-bib-0011] Gibbons, S. , Mourato, S. , & Resende, G. M. (2014). The amenity value of English nature: A hedonic price approach. Environmental and Resource Economics, 57, 175–196.

[cobi70061-bib-0012] Gorzula, S. (1996). The trade in dendrobatid frogs from 1987 to 1993. Herpetological Review, 27(3), 116–123.

[cobi70061-bib-0013] Harrison, J. R. , Roberts, D. L. , & Hernandez‐Castro, J. (2016). Assessing the extent and nature of wildlife trade on the dark web: Wildlife trade on the Dark Web. Conservation Biology, 30(4), 900–904. 26918590 10.1111/cobi.12707

[cobi70061-bib-0014] Hinsley, A. , Verissimo, D. , & Roberts, D. L. (2015). Heterogeneity in consumer preferences for orchids in international trade and the potential for the use of market research methods to study demand for wildlife. Biological Conservation, 190, 80–86.

[cobi70061-bib-0015] Hughes, A. C. (2021). Wildlife trade. Current Biology, 31(19), R1218–R1224. 34637735 10.1016/j.cub.2021.08.056

[cobi70061-bib-0016] International Union for Conservation of Nature (IUCN) . (2022). IUCN Red List of Threatened Species . https://www.iucnredlist.org/en

[cobi70061-bib-0017] Kaczmarski, M. , & Kolenda, K. (2018). Non‐native amphibian pet trade via Internet in Poland. European Journal of Ecology, 4(1), 30–40.

[cobi70061-bib-0018] Krishna, V. V. , Darras, K. , Grass, I. , Mulyani, Y. A. , Prawiradilaga, D. M. , Tscharntke, T. , & Qaim, M. (2019). Wildlife trade and consumer preference for species rarity: An examination of caged‐bird markets in Sumatra. Environment and Development Economics, 24(4), 339–360.

[cobi70061-bib-0019] Lyons, J. A. , & Natusch, D. J. D. (2013). Effects of consumer preferences for rarity on the harvest of wild populations within a species. Ecological Economics, 93, 278–283.

[cobi70061-bib-0020] Mohanty, N. P. , & Measey, J. (2019). The global pet trade in amphibians: Species traits, taxonomic bias, and future directions. Biodiversity and Conservation, 28(14), 3915–3923.

[cobi70061-bib-0021] Nijman, V. , & Shepherd, C. R. (2010). The role of Asia in the global trade in CITES II‐listed poison arrow frogs: Hopping from Kazakhstan to Lebanon to Thailand and beyond. Biodiversity and Conservation, 19(7), 1963–1970. 10.1007/s10531-010-9814-0

[cobi70061-bib-0022] Núñez, J. , Martín‐Barroso, D. , & Velázquez, F. J. (2024). The hedonic price model for the wine market: A systematic and comparative review of the literature. Agricultural Economics, 55, 247–264.

[cobi70061-bib-0023] Price, E. O. (1984). Behavioral aspects of animal domestication. The Quarterly Review of Biology, 59(1), 1–32.

[cobi70061-bib-0024] Roberts, D. L. , Mun, K. , & Milner‐Gulland, E. J. (2022). A systematic survey of online trade: Trade in Saiga antelope horn on Russian‐language websites. Oryx, 56(3), 352–359.

[cobi70061-bib-0025] Slone, T. , Orsak, L. , & Malver, O. (1997). A comparison of price, rarity and cost of butterfly specimens: Implications for the insect trade and for habitat conservation. Ecological Economics, 21(1), 77–85.

[cobi70061-bib-0026] Sung, Y.‐H. , & Fong, J. J. (2018). Assessing consumer trends and illegal activity by monitoring the online wildlife trade. Biological Conservation, 227, 219–225.

[cobi70061-bib-0027] Thompson, R. M. , Hall, J. , Morrison, C. , Palmer, N. R. , & Roberts, D. L. (2021). Ethics and governance for internet‐based conservation science research. Conservation Biology, 35(6), 1747–1754. 34057267 10.1111/cobi.13778

[cobi70061-bib-0028] Yeager, J. , & Zarling, A. (2020). Mediating ethical considerations in the conservation and sustainable biocommerce of the jewels of the rainforest. Journal for Nature Conservation, 54, Article 125803.

[cobi70061-bib-0029] Yeung, P. (2020). How mail‐order frogs could save Colombia's amphibians . https://www.bbc.com/future/article/20201216‐how‐mail‐order‐frogs‐could‐save‐colombias‐amphibians

