# Supplementary material for: Analysis of morphological attributes as a driver of trade in poison dart frogs
Source: Conserv Biol. 2025 May 31;39(5):e70061. doi: 10.1111/cobi.70061 (PMC12451504; doi:10.1111/cobi.70061)
Supplement: Supplementary file 1 — Appendix S1 – Summary of online data collected (n=420) [file COBI-39-e70061-s003.pdf]

Appendix S1 – Summary of online data collected (n=420)

Online data	Categories	No. of data entries
**Species**	*Dendrobates auratus*	141
	Dendrobates leucomelas	54
	Dendrobates tinctorius	223
	Dendrobates truncatus	2
**Country sold**	Belgium	14
	Canada	11
	United Kingdom	65
	United States	330
**Company/Platform**	Backwater Reptiles	4
	Black Jungle Exotics	43
	Dartfrog	4
	Dartfrog Connection	7
	Fantastic Frogs and Mythical Creatures	25
	Frog Daddy	34
	Hobby Reptiles	14
	JL – Exotics	10
	Josh’s Frogs	163
	Phelsuma Farm	1
	Preloved	17
	Reptile Rapture	7
	Reptiverse – Reptile Marketplace	2
	Snakes at Sunset	7
	South Coast Dart Frogs	12
	SwellReptiles	4

	Tails and Scales	11
	TCS Dart Frogs	12
	The Serpentarium	2
	Tincman Herps	11
	Toadally Frogs LLC	30
**Origin**	Captive bred	377
	Not stated	43
**Age**	Juvenile	243
	Froglet	47
	Adult	42
	Not stated	88
