# Supplementary material for: Analysis of morphological attributes as a driver of trade in poison dart frogs
Source: Conserv Biol. 2025 May 31;39(5):e70061. doi: 10.1111/cobi.70061 (PMC12451504; doi:10.1111/cobi.70061)
Supplement: Supplementary file 2 — Appendix S2 – Primary and secondary trunk colour combinations (n=420) [file COBI-39-e70061-s001.pdf]

Appendix S2 – Primary and secondary trunk colour combinations (n=420)

Colour Combinations	No. of data entries
Black and Blue	46
Black and Green	62
Black and Blue	17
Black and None	8
Black and White	19
Black and Yellow	110
Blue and Black	41
Blue and Brown	3
Brown and Blue	16
Brown and Cream	3
Brown and Green	22
Cream and Brown	3
Green and Black	9
White and Brown	11
Yellow and Black	43
Yellow and None	7
