# Supplementary material for: Analysis of morphological attributes as a driver of trade in poison dart frogs
Source: Conserv Biol. 2025 May 31;39(5):e70061. doi: 10.1111/cobi.70061 (PMC12451504; doi:10.1111/cobi.70061)
Supplement: Supplementary file 3 — Appendix S3 – Hedonic regression on the entire data set [file COBI-39-e70061-s002.pdf]

Appendix S3 – Hedonic regression on the entire data set

Variable	Coefficient	Standard error	p-value
Intercept	94.247***	5.506	<0.001
Europe	-1.265	3.746	0.736
Rarity	-0.462**	0.153	<0.01
HeadCol	11.928**	4.304	<0.01
Black	-0.472	3.298	0.886
Common	-16.168***	4.497	<0.001
LimbCol	-0.390	3.595	0.914
Rings	9.764	5.379	0.070
Spots	-4.812	3.761	0.202
Stripes	-2.906	3.956	0.463
Adjusted R^2	0.068		
F-statistic	4.412		<0.001

***, **, and * correspond to <0.001, <0.01, and <0.05 respectively.
